# Reflecting upon Similarities and Differences among the IAT, the IRAP, and the FAST: Searching for Clarity

**DOI:** 10.1007/s40614-025-00466-0

**Published:** 2025-07-09

**Authors:** Alceu Regaço, Colin Harte, Dermot Barnes-Holmes, Julio C. de Rose

**Affiliations:** 1 Universidade Federal de São Carlos, São Carlos, Brazil; 2 Instituto Par—Ciências do Comportamento, São Paulo, Brazil; 3 Instituto Nacional de Ciência e Tecnologia sobre Comportamento, Cognição e Ensino, São Carlos, Brazil; 4 Ulster University, Coleraine, Northern Ireland UK

**Keywords:** IAT, IRAP, FAST

## Abstract

The development of procedures to assess the psychological functions of stimuli has long been a prevalent endeavor in psychological science. It can be argued that one of the most influential of such procedures is the implicit association test (IAT), which was conceived as a measure of implicit attitudes within social-cognitive psychology. Two other procedures, developed within a behavior-analytic theoretical framework, were inspired by the IAT: the implicit relational assessment procedure (IRAP) and the function acquisition speed test (FAST). The current article aims to thoroughly describe and compare the IAT, the IRAP and the FAST, focusing on their procedural characteristics and theoretical backgrounds, before then considering theoretical implications specific to each procedure. Finally, we briefly reflect on a number of theoretical implications that arose from the foregoing comparisons. In so doing, several points of contact and departure are highlighted. We conclude by advocating for an equitable and comprehensive evaluation of these methodologies when considering their use within the experimental analysis of human behavior.

A fundamentally important question in psychological science concerns how a relatively neutral stimulus acquires a particular behavioral function. In the famous research by Ivan Pavlov, a neutral stimulus, such as a tone, was presented to dogs shortly before the delivery of food. At first, the dog did not salivate upon hearing the tone, but following multiple exposures to the contingent and temporal *relation* (i.e., tone-food pairings), the tone alone started to produce salivation (Pavlov, 1949). In effect, a new behavioral function had been established for the tone, based on the tone–food relation, using a procedure typically referred to as Pavlovian or classical conditioning. The acquisition of a novel function for the tone may be inferred based on the absence of salivation before the tone–food pairings and the observation of salivation after such pairing. In this case, therefore, the novel function is established in the laboratory. However, there are many instances in which psychology researchers may be interested in assessing or measuring the behavioral functions of stimuli that were likely established before an individual participated in a particular research project (e.g., Hagopian et al., 2004; Kang et al., 2013). In such cases, for example, a participant might be offered a choice among a number of food items to determine the individual’s preference for a particular food. Or, an individual might simply be required to rate how much they like each of the food items. In adopting this approach, the researcher is less focused on how the food preference was actually acquired, and more on ascertaining the food preference itself.

The history of psychology is replete with various methods and procedures for assessing the psychological functions of stimuli and events in an individual’s environment (e.g., the Likert scale; Jebb et al., 2021). As noted above, preference assessments and different rating scales have been used in this regard. In addition, psychological researchers have also developed a wide range of experimental procedures designed to test for the behavioral functions of specific stimuli. Many of these procedures typically involve asking participants to respond to stimuli in one context on some trials, and to respond to the same stimuli in a different context on other trials. The participant’s performance across these two contexts is then compared, thereby allowing the researcher to make certain inferences about the behavioral functions of the stimuli.

One of the most ubiquitous and well known of such procedures to emerge in recent decades is the implicit association test (IAT), a computerized procedure developed in the late 1990s with the aim of providing an indirect measure of attitudes (Greenwald et al., 1998, 2022). The basic premise of the IAT is that it should be easier to respond in a similar way to two stimuli that are more closely associated in memory than two stimuli that are less so (Greenwald & Banaji, 1995). As an example, research has found that participants tend to respond more quickly on the same response key to “flower” and “pleasant” than when they are required to respond on the same key to “insect” and “pleasant.” This difference in the response speed is taken to indicate that “flower” and “pleasant” are more closely associated in memory than “insect” and “pleasant.”

Of course, the basic idea that responding similarly to associated stimuli is easier and faster than responding similarly to not-associated stimuli predates the IAT (see Meyer & Schvaneveldt, 1971). Nevertheless, the IAT became pervasive across a wide range of research domains in psychology. For example, in a review of indirect measures frequently used from 2014 to 2018, the seminal IAT article alone (Greenwald et al., 1998) was cited over 2,100 times (Greenwald & Lai, 2020). Indeed, the IAT has been used in the study of topics ranging from racial bias (e.g., Ahadinezhad et al., 2022), to climate change (e.g., Fiorenza et al., 2023), and suicidal ideation (e.g., Sohn et al., 2021), to name but a few.

Although the behavior-analytic tradition would typically eschew the mentalistic theorizing linked to the IAT (i.e., that the IAT is a measure of associations in memory), a number of behavioral researchers did offer functional-analytic interpretations of effects produced on the measure (e.g., Barnes-Holmes et al., 2004; Barnes-Holmes & Harte, 2022b; O’Reilly et al., 2015; O’Toole et al., 2007). Furthermore, two specific procedures were developed within behavior analysis, which were inspired, in part, by the IAT: the implicit relational assessment procedure (IRAP; Barnes-Holmes et al., 2006) and the function acquisition speed test (FAST; O’Reilly et al., 2012). Much has been written about these three procedures in the behavioral-analytic literature, and some authors have considered a number of similarities and differences both theoretically (e.g., Barnes-Holmes et al., 2006; McKenna et al., 2007; O’Reilly et al., 2012, 2015; Power et al., 2009; Watters et al., 2023), and experimentally (e.g., Barnes-Holmes et al., 2009, Barnes-Holmes, Murtagh, Barnes-Holmes et al., 2010; Chan et al., 2009; da Silva et al., 2024; Farrell et al., 2015; Hussey et al., 2015; Roddy et al., 2010, 2011; van der Kaap-Deeder et al., 2018). However, there has been no comparison of the IAT, the IRAP, and the FAST in a *formal* and *systematic* way. The primary purpose of this article is to provide a detailed treatment of the three procedures, and in doing so, furnish the behavior-analytic research community with a useful reference point for determining the precise similarities and differences among them. Before continuing, we should be clear that it is not our aim to provide a systematic review or meta-analysis of the research using the IAT, the IRAP and the FAST. Rather, the article will consider a number of conceptual and empirical issues that potentially arise from points of contact and departure among the three procedures as experimental methods. We will first describe each procedure in turn, before comparing and contrasting them as procedures. We will then consider the main conceptual and theoretical issues associated with each procedure, before again comparing and contrasting them in terms of those issues.

## The IAT

**Fig. 1:**
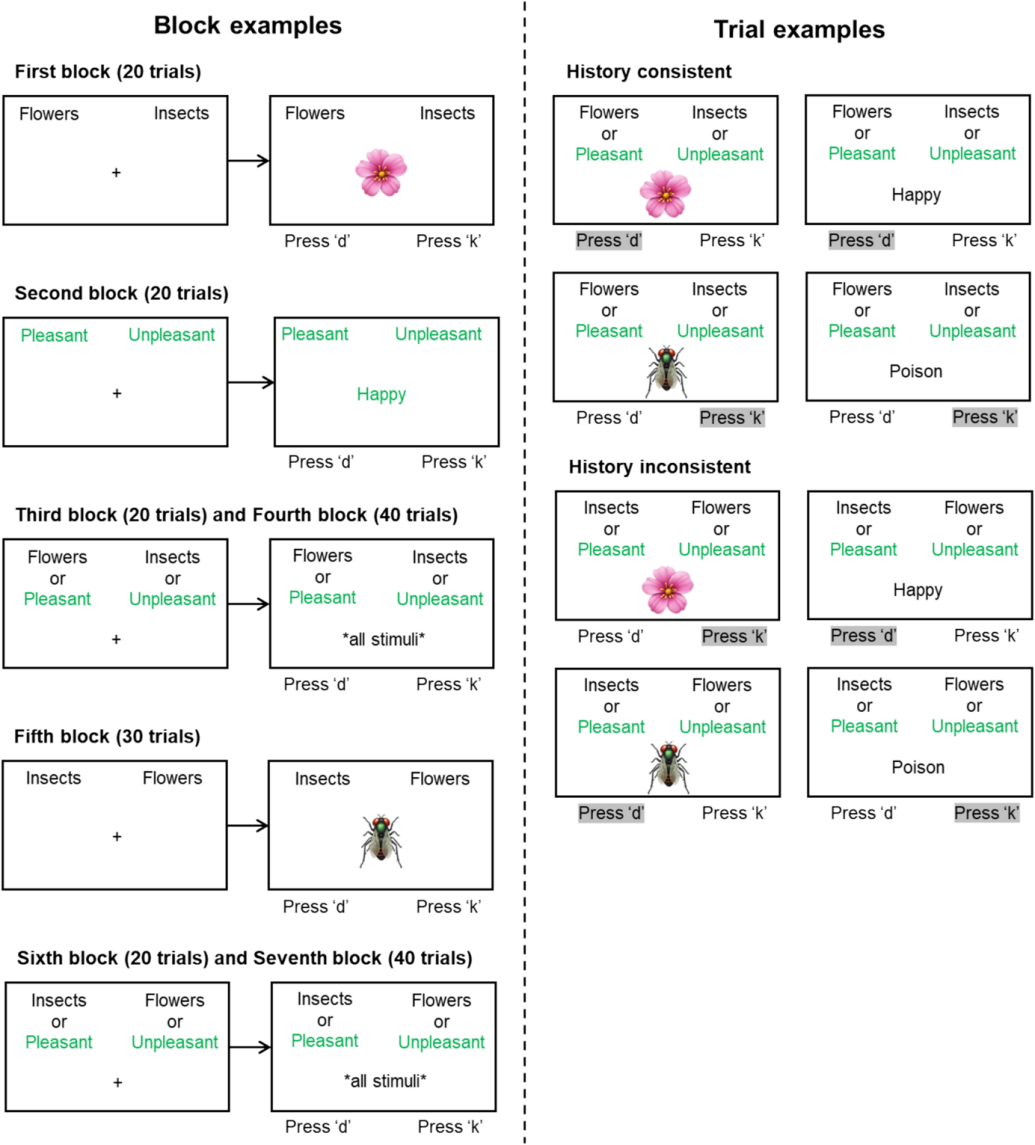
Block and Trials Representation of the IAT. *Note.* Correct selection for each trial example is highlighted in grey

The IAT is a computerized procedure that consists of seven blocks of trials, each block comprised of different numbers of trials and different numbers of stimuli (Greenwald et al., 2022). In a typical procedure, two categories of stimuli are employed: target stimuli (e.g., words “Flowers” and “Insects,” two nouns), and attribute stimuli (e.g., words “Pleasant” and “Unpleasant,” two adjectives). In a standard procedure, the first block (typically consisting of 20 trials) presents the target category. These appear in fixed positions in the upper corners of the screen, and a single stimulus (e.g., a picture or the name of a flower, or a picture or the name of an insect) is presented in the center of the screen (see Fig. 1). The participant is required to press one of two keys on the keyboard (one on the left and one on the right), which indicates whether the stimulus is an example of a Flower or an Insect. In the case of Fig. 1 (top panel), pressing the key on the left (flower) would be correct, whereas pressing the key on the right (insect) would be wrong.

In the second block (typically consisting of 20 trials), stimuli from the attribute category are presented in the upper corners of the screen, and a single stimulus (e.g., the word “Happy” or “Rotten”) is presented in the center of the screen. Once again, participants are required to indicate whether the stimulus is an example of “Pleasant” or “Unpleasant.” The third block (20 trials) is a training block, and it presents both target and attribute categories in the upper corners (e.g., “Flowers or Pleasant” in one corner, and “Insects or Unpleasant” in the other), and on each trial a stimulus is presented in the center of the screen from one of the four categories of stimuli presented in previous blocks (e.g., words with positive and negative valence, and pictures of flowers and insects). In this case, participants are required to indicate whether the stimulus is an example of “Flowers or Pleasant” or “Insects or Unpleasant.” The fourth block is identical to the third, but with more trials (e.g., 40) and it is described as a testing block. The fifth block is identical to the first block, but with the target stimuli in opposite positions, and typically with more trials; participants are now required to adjust their responses by pressing the key on the right for “Flowers” and the key on the left for “Insects.” The sixth and seventh blocks are identical to the third and fourth (they are also categorized as training and testing blocks, respectively), but with the target stimuli in the new positions. Across these two blocks, therefore, participants must press the left key for “Insects or Pleasant” and the right key for “Flowers or Unpleasant.” Thus, blocks 3 and 4 require participants to categorize "Flowers" with "Pleasant" (i.e., to respond to both with the same key) and “Insects” with “Unpleasant,” whereas blocks 5 and 6 require them to categorize "Flowers" with "Unpleasant" and "Insects" with "Pleasant." The basic idea is that participants should find it easier to press the correct key when categorizing “Flowers” with “Pleasant” and “Insects” with “Unpleasant” than when categorizing “Flowers” with “Unpleasant” and “Insects” with “Pleasant. As such, they would be expected to respond more quickly across blocks 3 and 4 than across blocks 6 and 7.

As noted above, on all trials, participants are required to press one of two keys, located on the left- and right-hand sides of the keyboard. Correct responses lead immediately to the presentation of an intertrial interval (e.g., 250 ms) whereas incorrect responses produce a red “X” just below the exemplar stimulus. Some IAT procedures require participants to respond correctly to complete the trial (if they make an error, they are required to press the other response key to continue), whereas other procedures register only the first response (independent of whether it is correct or incorrect). In addition, in the combined-task blocks (blocks 3, 4, 6, and 7), the procedure alternates across trials between target and category stimuli (e.g., if a stimulus on trial 1 is a flower or an insect, the stimulus on the next trial will either be a pleasant or unpleasant word). Furthermore, the side to which each category was assigned in the first block is typically counterbalanced across participants (e.g., if “Flower” was on the left for the first participant, it would be on the right for the second participant). Finally, half of the participants will typically receive the sequence of blocks described above and illustrated in Fig. 1, whereas the other half will receive a sequence in which the blocks containing incongruous categories (e.g., “Pleasant” with “Insect”) are presented before the blocks containing congruous categories (see Greenwald et al., 2022, for a detailed description of best practices when using the IAT).

### Data Analysis

The data selected for analysis are from a participant’s performance on blocks 3, 4, 6, and 7. If a participant produces latencies below 300 ms on more than 10% of trials (in the selected blocks), their whole dataset is excluded. Furthermore, individual latencies longer than 10 s are excluded from analysis (some studies also exclude trials with responses faster than 400 ms; see Greenwald et al., 2003, for a review of different types of data analysis). The most common way to calculate an overall score from the IAT involves calculating the two mean latencies from blocks 3 and 6 and then subtracting the former from the latter (this would be reversed if the incongruous blocks were presented before the congruous blocks). The resulting difference between the two means is then divided by the standard deviation calculated across those two blocks, yielding one D-score. The same calculation is applied to blocks 4 and 7, yielding a second D-score. The separate D-scores are then summed and divided by 2, yielding a final overall D-score for the participant’s performance. Greenwald et al. (2022) also describe two types of latency penalties that may be applied to trials on which participants produced an incorrect response. The first of these simply involves recording the latency until the first correct response (i.e., if the participant makes an error, the timer continues until the participant presses the correct key). The second penalty involves increasing the latency of the incorrect response by a certain amount (e.g., 600 ms). The authors indicate that the former practice is preferred.^1^ Finally, Greenwald et al. (2022) suggest that “. . . it is neither necessary nor desirable to drop subjects with relatively high error rates (even approaching 40%), so long as they are taking the procedure seriously and are trying to produce correct responses” (p. 1172). As such, a large number of errors are typically permitted when the IAT is employed in research.

### The FAST

**Fig. 2:**
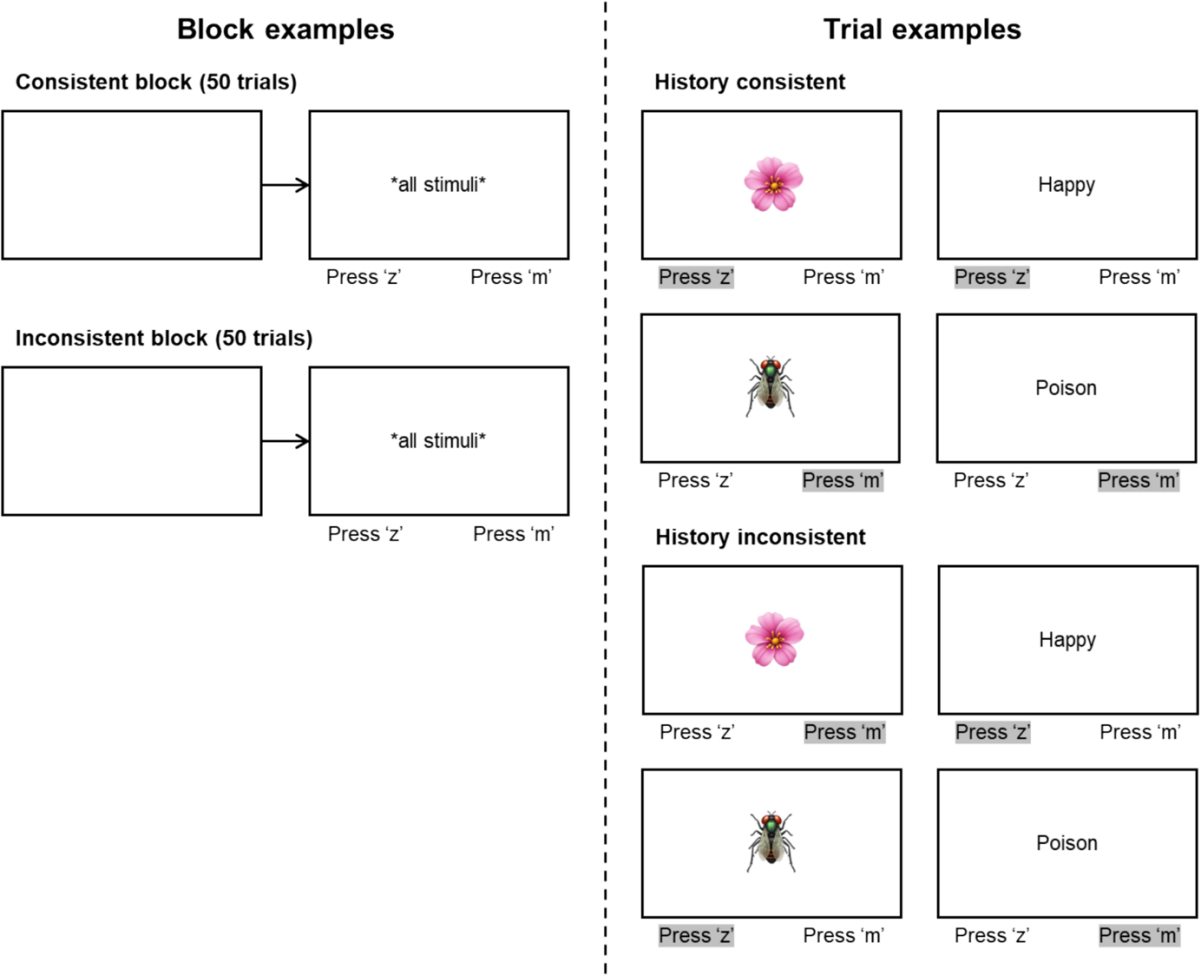
Visual Representation of Trials for Consistent and Inconsistent Blocks of the FAST. *Note.* Correct selection for each trial example is highlighted in grey

The FAST is a computerized procedure that, in its current version, consists of two blocks (consistent and inconsistent), typically involving 50 trials each. On all trials, a single stimulus is presented in the center of a blank screen (Figure 2) and participants must press one of two keys on the left or the right side of the keyboard (e.g., “d”/“k” or “z”/“m”). Similar to the IAT, two categories of stimuli are presented to participants: attribute stimuli, usually positive and negative words, and category stimuli, usually labels or traits (i.e., the target stimuli in the IAT). In the consistent block, participants must respond to stimuli that are coordinate on some basis using the same key on the keyboard (e.g., press “z” when “flowers” or “pleasant” are presented and press “m” when “insects” or “unpleasant” are presented). In the inconsistent block, participants must respond to stimuli that are distinct on some basis (e.g., press “z” when “insects” or “pleasant” are presented and press “m” when “flowers” or “unpleasant” are presented). Correct responses are followed by positive feedback (e.g., the word “correct” presented for 500 ms) and incorrect responses are followed by negative feedback (e.g., the word “wrong” for 500 ms). In addition, participants must respond within 3 s, otherwise negative feedback is presented (in the FAST literature, a failure to respond within 3 s is defined as an error). Following the feedback for correct or incorrect responding, an intertrial interval (e.g., 500 ms) is presented, followed by the next trial. FAST researchers also emphasize randomizing the order of presentation of the consistent and inconsistent blocks across participants.

### Data Analysis

Considering that the FAST is described as an acquisition test, there is no distinction between practice and testing blocks (earlier FAST research sometimes employed practice blocks but using stimuli unrelated to the stimuli used in the critical acquisition test). Participant performance is analyzed by comparing the number of correct responses in each of the two blocks. In particular, the number of cumulative correct responses is plotted against the cumulative number of trials, thus generating a learning curve. In more recent research, FAST analyses have involved calculating a regression line based on the learning curve for each block, and the slope of the inconsistent block is subtracted from the slope of the consistent block. A positive score indicates that the participant made fewer errors on the consistent than inconsistent block across the combined 100 trials. A negative score indicates the opposite (i.e., fewer errors on the inconsistent than consistent block). Therefore, for the FAST, and in contrast to the IAT, the data of interest is the number of correct responses in the consistent relative to the inconsistent block, rather than the response latency. On balance, as noted above, the procedure imposes a time limit for responses to occur (i.e., 3 s), and thus there is a temporal parameter to the FAST, which may affect the results arising from the learning curve produced by individual participants. Of course, a researcher would, in principle, be free to remove the data for those trials on which a participant failed to respond within the 3-s window.

### The IRAP

**Fig. 3:**
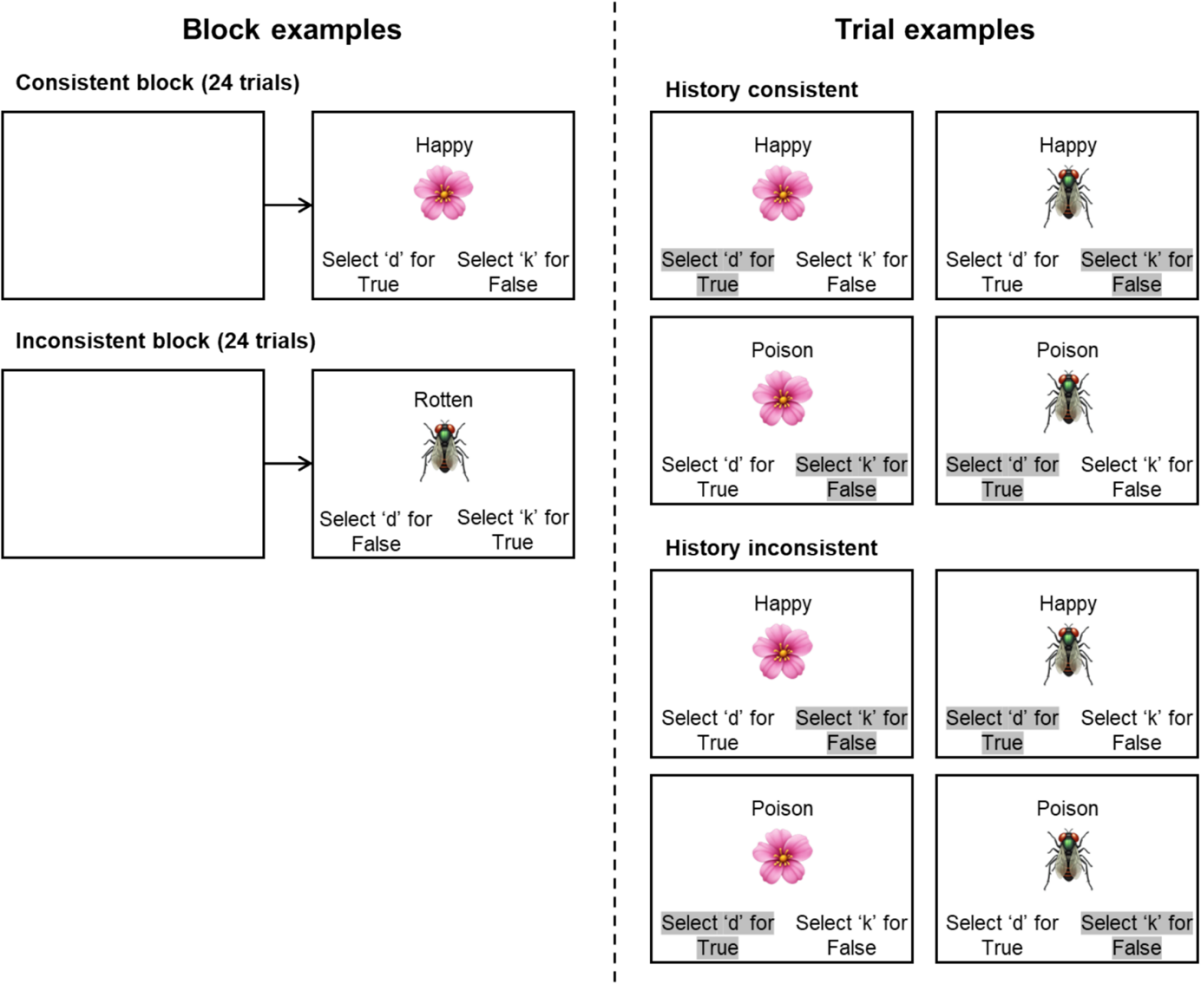
Trials Representation for Consistent and Inconsistent Blocks of the IRAP. *Note.* Correct selection for each trial example is highlighted in grey

The IRAP is a computerized procedure that consists of two types of blocks that present four types of trials. On all trials, one label (e.g., “flower” or “insect”) and one target stimulus (e.g., “pleasant” and “unpleasant”) are presented on the screen (the label is typically presented above the target stimulus, see Fig. 3). The four types of trials are generated by a 2X2 crossover of label and target stimuli. Following on from the previous example, the four trial-types would be flower-pleasant, flower-unpleasant, insect-pleasant, and insect-unpleasant. In addition, two response options are presented in the lower right- and left-hand corners of the screen, with an instruction for the participant to select one of two keys (e.g., “select ‘d’ for True” and “select ‘k’ for False”). The same four trial types are presented in all blocks (the minimum number of trials in any given block is typically 24). In consistent blocks, participants are required to respond in a pattern that coheres with an experimentally or pre-experimentally established learning history (e.g., when “flowers” and “pleasant” are presented, then responding “True” is deemed correct; when “insects” and “pleasant” are presented, then responding “False” is deemed correct). In inconsistent blocks, participants are required to do the opposite (e.g., when “flowers” and “unpleasant” are presented, then responding “True” is deemed correct; when “insects” and “unpleasant” are presented, then responding “False” is deemed correct). Correct responses result in the presentation of an intertrial interval (400 ms) followed immediately by the next trial. Incorrect responses result in the presentation of a red “x” below the target stimulus; participants are required to respond correctly to conclude the trial. In addition, participants are typically required to respond in less than 2 s, although the trial continues thereafter. In some IRAP studies, when the 2-s time window is exceeded, latency feedback is presented below the target stimulus (e.g., a red exclamation mark or the words “too slow”).

In each IRAP, participants are initially presented with a minimum of two practice blocks, consisting of pairs of consistent-inconsistent or inconsistent-consistent blocks (the order is sometimes randomized across participants) until they reach the mastery criteria. These criteria are often set at ≥ 80% correct responses and a mean response latency of ≤ 2 s, calculated within each block. These criteria must be reached for a pair of blocks to complete the practice phase (a maximum of four pairs of practice blocks is usually presented before the program terminates; a participant may be given the opportunity to repeat the practice phase immediately or at some later time if they so wish). After reaching the criteria, participants are presented with the testing phase, typically consisting of three pairs of blocks in the same order they were presented in the practice phase (i.e., the consistent and inconsistent blocks are always alternated). After every block, feedback is presented to the participant with the percentage of correct responses and the mean latency produced on the last block. In a typical procedure, the order in which the four trial-types are presented within each block is randomized; furthermore, the left/right location of the two response options (e.g., “True” and “False”) may be randomized across trials in each block.

### Data Analysis

The data from participants who reach the criteria in the practice phase and in all blocks of the testing phase are selected for analysis. In some studies, if a participant fails to maintain the criteria in one of the three block pairs, the data for that pair are removed from subsequent analyses (if they fail to maintain the criteria in two of the three block pairs, all data from that participant are discarded). The latencies from the IRAP are transformed into D-scores in a similar manner to that of the IAT, except four individual D-scores are calculated, one for each of the four trial types. Furthermore, unlike the IAT, only data from the test blocks are used (data from the practice blocks in the IAT are typically used to calculate D-scores). Similar to the IAT, if 10% of response latencies are less than 300 ms, all the data for that participant are excluded; any latencies larger than 10 s are also excluded. Note that in practice, however, these criteria would rarely apply because of the latency (≤ 2 s) and accuracy (≥ 80%) criteria that are applied at the block level (and sometimes even at the level of the trial-type; see de Almeida et al., 2024). As noted in note 1, with reference to the IAT, the latencies for error trials could also be removed with the IRAP, although the impact on the data would likely be much less for the latter because of the relatively high accuracy criterion generally employed, relative to the IAT.

Following the removal of data based on the criteria outlined above, the mean response latency for each of the four trial-types is calculated separately for each of the consistent and inconsistent blocks, yielding a total of 24 mean latencies. Mean latencies for each trial-type from consistent blocks are subtracted from mean latencies from inconsistent blocks, yielding 12 difference scores, and each difference score is divided by its associated standard deviation, calculated across the block pair. At this point, there are three standardized difference scores for each trial-type, one calculated across blocks 1 and 2, one calculated across blocks 3 and 4, and one calculated across blocks 5 and 6. These three difference scores for each trial-type are then averaged, yielding four separate trial-type D-IRAP scores (see Barnes-Holmes, Murtagh, Barnes-Holmes et al., 2010, for a detailed description of the data analysis process). A positive D-IRAP score indicates that the participant responded faster in consistent relative to inconsistent blocks, and a negative score indicates the opposite (i.e., faster in the inconsistent relative to the consistent blocks).

Before continuing, it is important to note that although the calculation of D-IRAP scores is by far the most common form of data analysis in IRAP research, alternatives are available. For example, some researchers have recommended the use of a semi-parametric probability index (De Schryver et al., 2018). In particular, this involves calculating the probability that latency scores in a consistent block for a particular trial-type will be less than the corresponding latency scores in an inconsistent block. Use of this probability index reduces the impact of outlying latency scores in terms of skew and kurtosis, issues typically associated with response-latency data.

## A Comparison between IAT, IRAP, and FAST

Having provided an overview of each of the three procedures, we will now consider the similarities and differences among them (see Table 1 for an overview), thus allowing us to reflect on conceptual and empirical issues arising from these points of contact and departure.

**Table 1:** Overall Characteristics of the IAT, the FAST, and the IRAP

	IAT	FAST	IRAP
**Maximum response time**	No	Yes	No
**Number of practice blocks**	5	No	≥ 2
**Criteria for practice blocks**	No	No	Yes
**Number of testing blocks**	2	2	6
**Criteria for testing blocks**	No	No	Yes
**Explicit feedback for correct responding**	No	Yes	No
**Explicit feedback for incorrect responding**	Yes	Yes	Yes
**Criteria for data analysis**	Yes	No	Yes

### Response Time

The FAST is the only procedure that requires participants to respond to the stimuli presented on the screen within a specific response window (3 s; see Table 1). That is, if participants fail to respond within 3 s, the procedure registers the trial as an incorrect response and continues to the intertrial interval. The IRAP and the IAT do not have a maximum response time criterion that actually terminates the trial. The IRAP does, however, present at the end of each block the participant’s mean response latency (and accuracy; see below) from that block. In addition, some research conducted with the IRAP presents feedback on every trial when a participant reaches the response time criterion (i.e., presents an “!” from 2 s). Therefore, although the IRAP does not have a maximum time limit for responding, participants are encouraged to respond within 2 s across all blocks, and their data are typically only included in subsequent analyses if they do so. The IAT is the only procedure that does not specifically impose a time limit for responding (by terminating the trial or requiring a mean response time across the block). However, the IAT applies a time limit criterion by excluding trials in which the response time is longer than 10 s and excluding the data for participants who responded faster than 300 ms in 10% or more of the trials (a procedure that is also employed with the IRAP).

### Practice Blocks

Both the IAT and the IRAP involve practice blocks whereas the FAST generally does not. In the IRAP, the practice blocks are similar to the testing blocks. In addition, the IRAP is the only procedure that requires participants to reach performance criteria in the practice blocks to proceed to the testing blocks. Therefore, the number of practice blocks is variable across participants, and all participants who reach the test blocks have met the accuracy and speed criteria in both consistent and inconsistent blocks. In the IAT, there are two practice blocks (blocks 3 and 6) that are identical to the testing blocks (blocks 4 and 7), and three practice blocks that present a similar but simpler task (blocks 1, 2, and 5; see Fig. 1 for details). IAT researchers frequently include data from practice blocks 3 and 6 in their analyses (i.e., combining them with data from the testing blocks). Furthermore, unlike the IRAP, no specific performance criteria are applied to the practice blocks; participants simply proceed directly from a practice block to the next block, irrespective of the participant’s performance. The FAST generally does not employ practice blocks, which appears to be consistent with its conception as a measure of response acquisition (rather than mastery). That is, allowing participants to practice the very performances the researcher is attempting to assess (i.e., acquisition) would, we presume, undermine the basis of the measure (i.e., the difference between the two types of blocks would be reduced or perhaps even eradicated). Finally, all procedures provide explicit feedback for incorrect responses, with the FAST being the only procedure that provides explicit feedback for correct responding.

### Test Blocks

Both the IAT and the FAST present one test block for consistent relations and one test block for inconsistent relations (yielding two testing blocks). The IRAP presents three test blocks for consistent relations and three test blocks for inconsistent relations, alternating between consistent and inconsistent, yielding a total of six testing blocks. The IRAP is the only procedure that requires participants to maintain the criteria for both accuracy and response latency across the testing blocks (for their data to be included in subsequent analyses). In addition, the IRAP is the only procedure that presents more than two test blocks in a sequence. That is, instead of presenting consistent-inconsistent or inconsistent-consistent once, it repeats one of these two sequences three times, requiring participants to alter the pattern of responding across multiple blocks.

### Data Analysis

The data analyses typically conducted with the IAT and the IRAP are similar to each other. That is, both produce a D score based on the response latencies for assessing the difference between responding to consistent versus inconsistent trials. The FAST is different from the IAT and the IRAP because the variable of interest is the number of *correct* responses for each consistent or inconsistent block. Therefore, the data analysis conducted by FAST researchers focuses on the cumulative number of correct trials and the slope of the curve it generates when plotted against the number of trials. Overall, data treatment conducted with all the three procedures is based on the variables targeted by the procedure (i.e., response latency for the IAT and the IRAP, and response accuracy for the FAST).

In any case, it is worth emphasizing that the IAT, the FAST, and the IRAP are not irreversibly wedded to any particular scoring method. Although IAT and IRAP data analyses are focused on response latency, in principle, performance on these procedures could be analyzed in terms of response accuracy during practice and/or test blocks (this information is available in the data). Researchers using the FAST could also analyze response latency for consistent and inconsistent trials (although response speed would of course be limited to the response window of 3 s).

## Emerging Conceptual Differences

Having considered the specifics of each procedure and the differences and similarities among them, we will now turn to how these procedures and the research conducted with them have resulted in different conceptual issues. Before doing so, it is important to note that although each of the procedures will be considered, they differ in relation to the amount of experimental and theoretical research available for analysis. On the APA PsycInfo database in October 2024, the search for “implicit association test” yielded 3,795 peer-reviewed publications, the search for “implicit relational assessment procedure” yielded 194 peer-reviewed publications, and the search for “function acquisition speed test” yielded 10 peer-reviewed publications. There are likely many other publications not included in those numbers, but it remains the case that there are huge differences among the procedures regarding their prevalence in the literature.

### Theoretical Implications of the IAT

The IAT was initially proposed as an implicit measure of attitudes, with the name “implicit” meaning that the phenomenon of interest was being assessed indirectly (Greenwald et al., 2022). That is, instead of asking participants to evaluate a certain subject (e.g., what do they think about flowers and insects), the researchers used the IAT as an indirect measure of the evaluation of that same subject (e.g., would they respond faster to flower/positive and insect/negative over flower/negative and insect/positive). Despite the apparent simplicity of the IAT, the definition of “implicit,” as well as what exactly the IAT assesses, is still a matter of debate among different researchers. In a review of the meaning of the concept “implicit,” Corneille and Hütter (2020) identified three broad theoretical views that are typically used to define what “implicit” means:

1. Implicit as the indirectness of the measure;
2. Implicit as automatic; and
3. Implicit as associative learning and representation.

The authors argued that the differences among the definitions of “implicit” generate significant confusion and they suggested abandoning the concept. In a similar vein, in a review of theoretical definitions of “implicit bias,” Brownstein et al. (2019, p. 9) concluded by advocating for a “behavioral approach that treats performance outcomes on implicit measures as *behaviors* rather than direct indicators of *mental constructs*” (emphasis added; see Barnes-Holmes & Harte, 2022b, for a broadly similar argument with respect to the IRAP).

There appear to be clear differences in the use of the concept “implicit” among researchers and among critics of that concept. On balance, a recent article on best practices for using the IAT concluded that “understandings of ‘implicit’ should not affect interpretation or application of the article’s conclusions about recommended research practices when describing the best practices to use the IAT” (Greenwald et al., 2022, p. 1162). That is, according to Greenwald et al., theoretical differences should not influence the use of the IAT as a procedure, suggesting that these differences may not be as important as reported by some authors. Overall, despite the multiple theoretical perspectives, research with the IAT has provided a basis for the development of a growing literature on implicit biases, with a focus on prejudice and stereotyping (Gawronski et al., 2020). This development has fostered research on several important applied topics (such as gender and racial biases, among many others), while also drawing attention from the general public to these questions. In addition, the IAT has been used in the behavior-analytic literature as a method for assessing the acquisition of stimulus functions via derived relations (O’Toole et al., 2007), and indeed inspired, in part, the development of a wide range of other procedures, including the IRAP and the FAST.

### Theoretical Implications of the FAST

A major theoretical basis for the development of the FAST was stimulus equivalence classes and functional response classes (O’Reilly et al., 2012). In stimulus equivalence research, after conditional discrimination training involving arbitrary sets of stimuli, human participants typically derive new relations that are not explicitly reinforced (Sidman & Tailby, 1982). For example, after training A to B and B to C, participants will typically match A to C and C to A without further training. Another characteristic of equivalence classes is the transfer of function across members of the same class: if one stimulus affects behavior (i.e., has a behavioral function), members of the same equivalence class will likely control behavior in a similar manner (Dougher et al., 1994; Sidman, 2000). Following on from the previous example, if A is established as a punisher through direct stimulus pairing, then both B and C may also acquire punishing functions in the absence of direct pairing. It is critical to note that research has also shown that a stimulus function established prior to a new experimentally trained stimulus relation may interfere with the derivation of equivalence classes (e.g., Leslie et al., 1993; Tyndall et al., 2004; Watt et al., 1991). For example, participants may be less likely to show equivalence class formation between flower stimuli and unpleasant stimuli relative to flower and pleasant stimuli.

According to Roche et al. (2005), such research within stimulus equivalence could be linked to the behavior assessed by the IAT. Indeed, the authors affirmed that “stimulus equivalence research may reveal some clues as to the core processes involved in IAT performances” (p. 116). In research aiming to test this hypothesis, Gavin et al. (2008) used a procedure similar to the IAT to test for class-consistent and class-inconsistent responding to stimuli that participated in equivalence classes. As a result, participants accuracy was higher for class-consistent over class-inconsistent responding, reinforcing the argument that stimulus equivalence may be relevant to the effects observed with the IAT. Within this broad context, the FAST may be seen as a means of assessing the extent to which stimuli are related when they share or do not share a common behavioral function. Indeed, a number of publications using the FAST assessed experimentally induced equivalence relations (e.g., Cummins et al., 2018; Cummins & Roche, 2020; O’Reilly et al., 2012, 2013; Passarelli et al., 2024). Overall, research with the FAST has provided a behavioral interpretation of the phenomenon of implicit bias based on the concept of stimulus equivalence, while also providing a means of assessing the strength of stimulus equivalence relations (Bortoloti & de Rose, 2011; Watters et al., 2023).

### Theoretical Implications of the IRAP

Development of the IRAP was based in part on the IAT, but with a focus on assessing specific relational responses rather than associations. A major difference between the two procedures, therefore, is that the IAT was not seen to specify the nature or directionality of the relations between the stimuli (De Houwer, 2002), whereas the IRAP was designed to provide more precision in this regard (Barnes-Holmes et al., 2006). The IRAP’s focus on relations rather than associations was grounded in relational frame theory (RFT; Hayes et al., 2001), and specifically in an RFT-based procedure, known as the relational evaluation procedure (REP; Hayes & Barnes, 1997). In fact, the IRAP, in a broad sense, involved combining the IAT with the REP (see Barnes-Holmes & Harte, 2022b); the resulting IRAP thus yielded data pertaining to specific relational responses rather than simply associations. This is reflected in the fact that, whereas the IAT yields one D-score, the IRAP yields four, one for each trial-type.

At first, these four trial-type D-scores were interpreted as indicating relational response strength. Simply put, the larger the D-score, the greater the strength of the response (Barnes-Holmes, Barnes-Holmes et al., 2010). In more recent research, however, this rather simplistic view has been replaced by a more complex and sophisticated functional interpretation of responding on the IRAP, known as the differential arbitrarily applicable relational responding effects (DAARRE) model (Finn et al., 2018, 2019). This more recent work seems to be producing theoretical advances in terms of understanding IRAP performances, and also within RFT itself (Barnes-Holmes et al., 2021; Barnes-Holmes & Harte, 2022a; Harte & Barnes-Holmes, 2024). In particular, these advances highlight potentially important differences between the semantic versus emotional/attentional properties of stimuli participating in derived relations (Harte, 2024). In addition, these conceptual advances go very much beyond what was initially proposed in the earlier IRAP literature (cf. Barnes-Holmes, Barnes-Holmes et al., 2010), and are generating a growing body of relevant empirical work (e.g., Bortoloti et al., 2023, 2024; Pidgeon et al., 2021; Pinto et al., 2020; Schmidt et al., 2021). As such, more recent research using the IRAP appears to be supplanting its previously dominant use in the study of implicit attitudes with a refocus on understanding human language and cognition from a behavior-analytic viewpoint, involving ongoing development both experimentally and theoretically (see Barnes-Holmes & Harte, 2022a, 2022b; de Almeida et al., 2024; Harte & Barnes-Holmes, 2024).

## Theoretical Comparison of the IAT, the IRAP, and the FAST

Having considered each one of these procedures, we will briefly reflect on their theoretical similarities and differences. The theoretical background for the development of the IAT was rooted in the mentalistic concepts of implicit associations in memory and automatic activation (Greenwald et al., 1998; Greenwald & Banaji, 1995; Greenwald & Lai, 2020). However, it has been argued that the relationship of these two concepts to the IAT and the behavior it assesses is not straightforward. The concept of implicit memory was developed in the context of studies examining the difference between different types of memory tasks (Graf & Schacter, 1985), and the concept of automatic activation was developed in the context of priming studies (Fazio et al., 1986). Indeed, in a review of the priming and the IAT literature, Fazio and Olson (2003) affirmed that “although IAT effects are often referred to as ‘automatic preferences,’ this use of the term automatic appears to have a very different meaning than it does in the context of priming procedures” (p. 315) and that “precisely how the IAT works remains unclear” (p. 314). As a consequence, the development of any formal model (e.g., ReAL; Meissner & Rothermund, 2013) that could be used to understand the psychological processes underlying the IAT must be retrospective (i.e., developed after the procedure). In contrast, the IRAP and the FAST were both developed with a previously established theoretical analysis within the behavior-analytic tradition. In particular, the IRAP was developed based on theoretical analyses grounded in RFT, and the FAST was developed based on stimulus equivalence research and theory. In any case, the lack of a formal theoretical model in the development of the IAT was likely one of the sources of the theoretical confusion that arose from it (Brownstein et al., 2019; Corneille & Hütter, 2020).

Another difference between the IAT, the FAST, and the IRAP is their focus on different parts of the behavioral stream as it occurs on the procedure. In particular, an IAT performance appears to be based on both acquisition (because most studies include the practice blocks into the data analysis), and on postacquisition behavior (because it involves test blocks following practice). A FAST performance, however, is based entirely on acquisition in that the participant is exposed to a fixed set of practice/training trials, and the data of interest involves the number of correct responses made during those trials. In contrast, the IRAP is largely based on postacquisition behavior because practice block data are not typically included in the analyses, and only data that meet specific levels of accuracy and response speed are considered. Therefore, at least theoretically, the procedures differ in terms of the part of the behavioral stream they are most focused on, and direct comparisons should always be made with caution, at least until the functional differences between acquisition and postacquisition performances can be better understood.

Finally, it can be argued that both the IAT and the FAST largely focus on a single concept to explain their effects, respectively, association and equivalence. The effects produced by each of the procedures are typically interpreted in terms of those concepts, associative strength in the IAT (Mandelbaum, 2016) and strength of equivalence relations in the FAST (O’Reilly et al., 2013; Watters et al., 2023). In contrast, the IRAP was generated within the context of RFT and thus, by definition, included the concept of multiple stimulus relations (e.g., similarity, opposition, comparison) and transformation of functions. In addition, more recent developments in terms of the DAARRE model have highlighted an important distinction between the RFT concepts of Crel and Cfunc stimulus properties (Barnes-Holmes & Harte, 2022b). In this regard, the IRAP is perhaps distinguished from the IAT and the FAST in that there appears to be ongoing theoretical development aimed at producing a broader understanding of human language and cognition generally, rather than implicit bias or the strength of equivalence relations in particular.

## Conclusion

The aim of this article was to describe the IAT, the IRAP, and the FAST in detail by focusing on and exploring their characteristics as procedures, and discussing their theoretical background and the issues they have raised. In doing so, we have argued that the three procedures have different characteristics, which may affect the behavior each assesses and the recorded data that is typically considered for analysis. In addition, the IAT, the IRAP, and the FAST were developed with different theoretical bases and different research foci (although there are some intersections with each other), ultimately resulting in different theoretical implications. As such it would be unwise to assume that each of the procedures necessarily captures the same behavioral processes. Indeed, as recent theoretical developments around the IRAP have highlighted, the behavioral processes at play within the procedure are relatively complex and require further empirical enquiry (e.g., de Almeida et al, 2024).

In conclusion, although there are some similarities, the IAT, the IRAP, and the FAST differ in many ways, which makes a direct comparison between or among them extremely difficult. At this point, therefore, it seems somewhat naïve to argue or conclude that one is “better” or “worse” than the other in any absolute sense (see Harte et al., 2024; Regaço et al., 2025, for recent literature advocating for a less combative approach within behavior analysis in general). In any case, any useful comparison, in our view, will depend largely on the research aims of each specific study, and perhaps more important, on the broad research agenda that is driving the use of the procedure. We hope that the description provided here will help other researchers to glean a better understanding of the IAT, the FAST, and the IRAP. In doing so, it was our aim to provide a relatively clear view of the similarities and differences among them, thus assisting in future research efforts, both empirical and theoretical, involving one or more of these procedures.

## Data Availability

Not applicable.

## Footnotes

1 As one reviewer pointed out, another option would be to simply remove the latencies on all of those trials in which errors occurred. Indeed, doing so might be preferred if the researcher is attempting to measure correct response fluency.
